# Targeting oncogenic Ras by the *Clostridium perfringens* toxin TpeL

**DOI:** 10.18632/oncotarget.24740

**Published:** 2018-03-27

**Authors:** Björn Schorch, Hannah Heni, Nour-Imene Zahaf, Tilman Brummer, Marina Mione, Gudula Schmidt, Panagiotis Papatheodorou, Klaus Aktories

**Affiliations:** ^1^ Institut für Experimentelle und Klinische Pharmakologie und Toxikologie, Medizinische Fakultät, Albert-Ludwigs-Universität Freiburg, Freiburg, Germany; ^2^ Institut für Molekulare Medizin und Zellforschung, Medizinische Fakultät, Albert-Ludwigs-Universität Freiburg, Freiburg, Germany; ^3^ German Cancer Consortium (DKTK), Partner Site Freiburg, Germany, and German Cancer Research Center (DKFZ), Heidelberg, Germany; ^4^ Centre for Biological Signalling Studies (BIOSS), Albert-Ludwigs-Universität Freiburg, Freiburg, Germany; ^5^ Institute of Toxicology and Genetics, Karlsruhe Institute of Technology, Eggestein-Leopoldshafen, Germany; ^6^ Present Address: Center for Integrative Biology, University of Trento, Trento, Italy; ^7^ Present Address: Institute of Pharmaceutical Biotechnology, University of Ulm, Ulm, Germany; ^8^ Present Address: Institute of Pharmacology and Toxicology, University of Ulm Medical Center, Ulm, Germany

**Keywords:** Clostridium perfringens toxin, glycosylation, immunotoxins, paradoxical activation, Ras

## Abstract

*Clostridium perfringens* toxin TpeL belongs to the family of large clostridial glycosylating toxins. The toxin causes N-acetylglucosaminylation of Ras proteins at threonine35 thereby inactivating the small GTPases. Here, we show that all main types of oncogenic Ras proteins (H-Ras, K-Ras and N-Ras) are modified by the toxin *in vitro* and *in vivo*. Toxin-catalyzed modification of Ras was accompanied by inhibition of the MAP kinase pathway. Importantly, TpeL inhibited the paradoxical activation of the MAP kinase pathway induced by the BRAF inhibitor Vemurafenib in the human melanoma cell line SBCL2. The toxin also blocked Ras signaling in a zebrafish embryo model expressing oncogenic H-Ras^G12V^, resulting in a reduction of melanocyte number. By using the binding and translocation component of anthrax toxin (protective antigen), the glucosyltransferase domain of TpeL was effectively introduced into target cells that were not sensitive to native TpeL toxin. To reach a higher specificity towards cancer cells, a chimeric TpeL toxin was engineered that possessed the knob region of adenovirus serotype 35 fiber, which interacts with CD46 of target cells frequently overexpressed in cancer cells. The chimeric TpeL fusion toxin efficiently inhibited Ras and MAP kinases in human pancreatic cancer Capan-2 cells, which were insensitive to the wild-type toxin. The data reveal that TpeL and TpeL-related immunotoxins provide a new toolset as Ras-inactivating agents.

## INTRODUCTION

Ras proteins, with their major isoforms H-, K- and N-Ras, are molecular switches, which are regulated by cycling between an inactive GDP and an active GTP-bound form [1–3]. They are controlled by guanine nucleotide exchange factors (GEFs; e.g. *son of sevenless* and others), which activate Ras proteins, and by GTPase activating proteins (GAPs, e.g., neurofibromin 1 and others), which terminate the activated state of Ras proteins [4]. Ras proteins are master regulators of proliferation, differentiation and survival processes by controlling several cellular signaling pathways, including the MAP kinase pathway cascade Raf-MEK-ERK [3, 5]. Activating mutations of *RAS* (oncogenic *RAS*) play pivotal roles in carcinogenesis and in tumor development [6]. While several kinase inhibitors of Raf, MEK or ERK proteins have been developed, which are successfully used as antitumor drugs, similar small molecular inhibitors of Ras proteins are not available [7]. However, Ras is a target of *Clostridium perfringens* toxin TpeL, which inactivates the switch protein by attachment of N-acetylglucosamine (GlcNAc) [8–10].

TpeL belongs to the family of large clostridial glycosylating toxins [11–14]. Prototypes of this toxin family are the Rho-glucosylating *C. difficile* toxins A and B, which are the cause of antibiotics-associated diarrhea and pseudomembranous colitis [14]. Similar to *C. difficile* toxins, TpeL consists of an N-terminal glucosyltransferase domain that is followed by an auto-protease domain and a domain involved in delivery of the toxin into target cells [8–10]. The C-terminal part of TpeL harbors a receptor binding region [15]. Recently, low-density lipoprotein receptor-related protein 1 (LRP1) was identified as a cell surface receptor of the toxin [15]. TpeL does not possess a C-terminal CROPs (combined repetitive oligopeptides) domain that is typical for all other clostridial glucosylating toxins and suggested to be involved in receptor binding.

TpeL entry into cells depends on various steps. It starts with the binding to LRP1 of host cells [15]. Subsequently, the toxin-receptor complex is endocytosed and translocated to low pH endosomal compartments. At low pH, the toxin undergoes conformational changes and inserts into the vesicle membrane. This allows translocation of the glycosyltransferase and cysteine protease domain into the cytosol, where inositol hexakisphosphate (InsP6) activates the protease activity, resulting in the release of the glucosyltransferase domain (GTD). In the cytosol, released GTD of TpeL causes GlcNAcylation of Ras proteins, using UDP-GlcNAc as a sugar donor. Modification of Ras occurs at threonine35, thereby the Ras protein is inactivated and Ras-dependent signaling pathways are blocked [10].

Here, we studied the action of TpeL on Ras in various types of tumor cells and in zebrafish embryos, expressing a hyperactive Ras mutant in melanocytes. We show that the toxin inhibits Ras-dependent signaling in tumor cells and in the zebrafish model, expressing activated Ras proteins. Moreover, we report that TpeL is capable of inhibiting the Raf-MEK-ERK pathway in *NRAS* mutant melanoma cells, which are paradoxically activated by the B-Raf kinase inhibitor Vemurafenib, indicating an essential role of Ras in this activation. Finally, we report on the construction of chimeric toxins of TpeL with the aim to increase the cell type selectivity of the Ras-inactivating *C. perfringens* toxin.

## RESULTS

### TpeL glycosylates oncogenic Ras *in vitro* and in cancer cells and counteracts the paradoxical MAP kinase activation by vemurafenib

**Figure 1:**
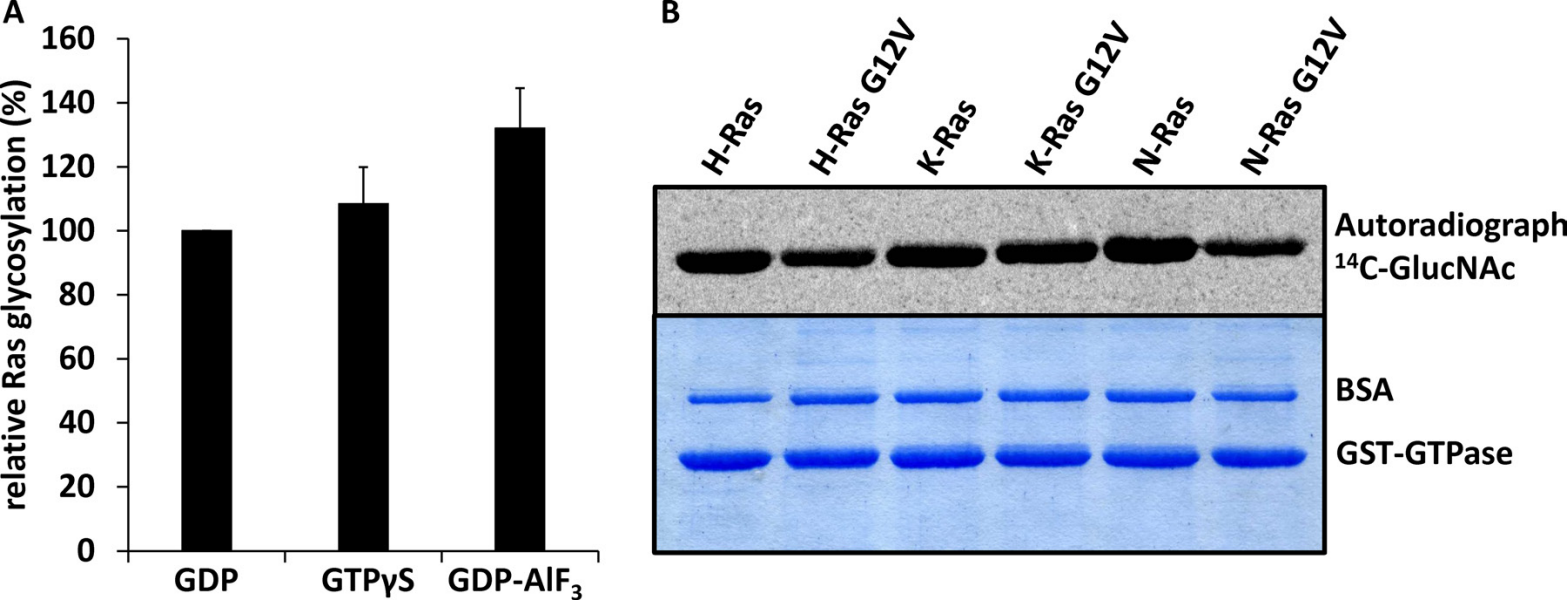
TpeL_GTD_ glycosylates Ras proteins irrespective of nucleotide binding and hyperactive mutations *in vitro* (**A**) Relative initial glycosylation velocities were calculated from three independent experiments for K-Ras preloaded with GDP, GTP or GDP-AlF_3_. (**B**) Wild type as well as G12V mutated K-, H-, and N-Ras proteins were glycosylated by TpeL_GTD_. Shown are the autoradiogram (upper panel) and the Coomassie-stained gel (lower panel).

At first, we studied the GlcNAcylation catalyzed by the glucosyltransferase domain of TpeL *in vitro* with Ras loaded with GDP, GTPγS and GDP plus aluminium trifluoride (AlF_3_) to mimic the active and inactive states of Ras. Under all conditions, Ras was efficiently modified by TpeL (Figure 1). Then, we analyzed the substrate properties of various Ras isoforms, including the common oncogenic G12V mutation, for GlcNAcylation by the GTD of TpeL. All isoforms and mutants were modified to a comparable extent, even if the relative initial reaction velocity of modification of N-Ras^G12V^ was reduced by about 40%.

**Figure 2:**
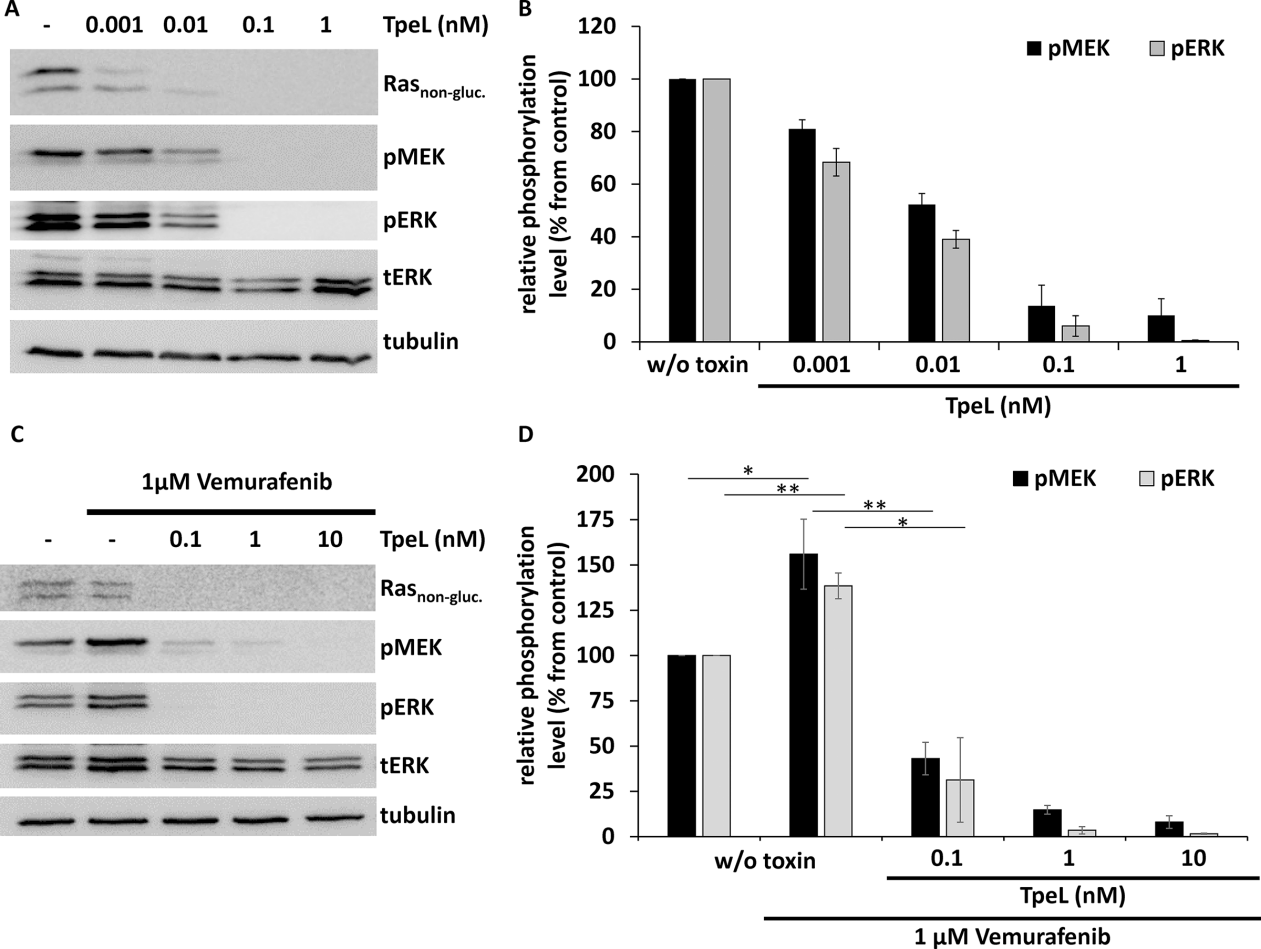
TpeL effects the oncogenic and paradoxical activation of MAPK signaling in SBCL2 cells (**A**) Western blot analysis of SBCL2 cells intoxicated with increasing concentrations of TpeL for 4 h. Ras_non-gluc._, pMEK, pERK, tERK, and tubulin were probed. A representative blot of three independent experiments is shown. (**B**) Statistical analysis of phosphorylated MEK and ERK following TpeL treatment as presented in A. (**C**) Western blot analysis of SBCL2 cells intoxicated with increasing concentrations of TpeL for 4 h following preincubation with Vemurafenib for 2 h. Ras_non-gluc._, pMEK, pERK, tERK, and tubulin were probed. A representative blot of three independent experiments is shown. (**D**) Statistical analysis of phosphorylated MEK and ERK following treatment with Vemurafenib and TpeL as presented in C.

Next, we studied the effect of TpeL holotoxin on the human melanoma cell line SBCL2. These cells represent a model for the initial (radial) growth phase of melanoma and harbor the *NRAS* Q61K mutation, but no *RAF* V600E mutation. Cells were intoxicated with increasing concentrations of TpeL for 4 h. Subsequently, cells were lysed and the extent of Ras modification was determined by an anti-Ras antibody that only interacts with non-modified Ras protein [16]. Moreover, the phosphorylation of MEK and ERK was determined by immunoblotting with specific antibodies. Figure 2A and 2B show that TpeL caused modification of Ras at subnanomolar concentrations. At 0.1 nM TpeL, phosphorylation of MEK and ERK was completely blocked.

Vemurafenib is a potent and specific kinase inhibitor of B-Raf V600E [17]. The compound is used as an antitumor drug, however, only in cases of the *BRAF* V600E mutation. It is well-known that in the presence of wild-type B-Raf and activated Ras, Vemurafenib paradoxically activates the MAP kinase pathway [18–20]. As described recently [21, 22], paradoxical activation of MEK and ERK by Vemurafenib (1 µM) was confirmed also in SBCL2 cells (Figure 2C and 2D). Importantly, treatment of SBCL2 cells with TpeL inhibited the paradoxical activation of MAP kinases by Vemurafenib in a concentration-dependent manner.

### TpeL inhibits Ras signaling in a zebrafish model of melanoma

**Figure 3:**
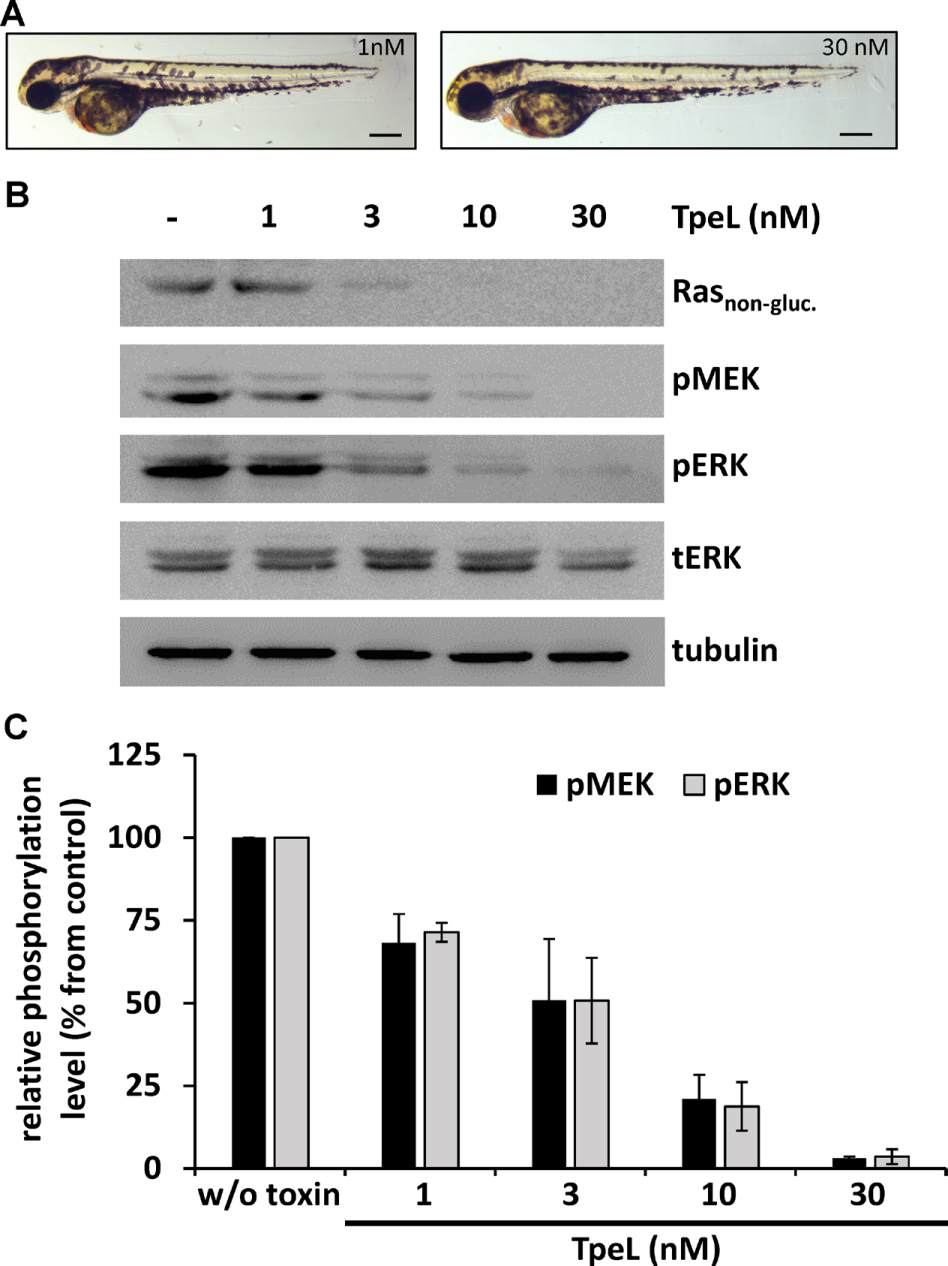
TpeL treatment reduces Ras-dependent hyperproliferation of melanocytes *in vivo* (**A**) Micrographs of zebrafish larvae with Ras-dependent hyperpigmentation at 60 hpf treated with low (1 nM) and high (30 nM) concentrations of TpeL from 12 hpf on. A reduced pigmentation in the high TpeL concentration treated fish is seen over the whole larvae. The scale bar represents 0.1 mm. (**B**) TpeL- or untreated zebrafish larvae were pooled to 20 embryos per condition and lysed. Subsequently, Ras_non-gluc._, pMEK, pERK, tERK, and tubulin were probed by immunoblotting. A representative blot of three independent experiments is shown. (**C**) Statistical analysis of phosphorylated MEK and ERK following treatment with TpeL as presented in B.

Santoriello and coworkers introduced a zebrafish model for melanocyte hyper-proliferation and melanoma by the expression of *HRAS* G12V under the control of the melanocyte-specific *kita* promoter [23]. These zebrafish embryos were used to evaluate the ability of TpeL to act on Ras-driven, hyper-proliferative cells *in vivo* and to assess tolerability for the whole organism. To this end, embryos were dechorionated at shield stage (6 hours post fertilization, hpf) and treated with TpeL (1, 3, 10, and 30 nM) from 12 to 60 hpf. Five days post fertilization larvae from the double transgenic line *hzm1Et;io006tg* showed a strong pigmented head region and a melanocyte cluster-rich tail. TpeL treatment of zebrafish embryos at concentrations ranging from 3 to 30 nM strongly reduced this pigmentation, with the higher doses leading to a normal number and distribution of melanocytes over the whole body (Figure 3A and Supplementary Figure 1).

To determine the direct effect of TpeL on MAPK signaling in zebrafish, whole larval lysates were probed by immunoblotting (Figure 3B and 3C). Additionally to Ras_non-gluc._, pMEK and pERK were strongly reduced in a concentration dependent manner by TpeL treatment. Most likely indicating an inactivation of upstream Ras by glycosylation with subsequent reduction in the number of melanocytes expressing hyperactive Ras.

All zebrafish were examined by 3 independent researchers at the end of the experiments before lysis. There were no signs of developmental failure or toxicity visible. Given the high sensitivity of microscopy screening using zebrafish embryos to detect toxic effects, this indicates for a strong tolerance of the organism towards TpeL in general (Supplementary Figure 1).

### Construction of TpeL chimeric toxins for cancer-specific targeting

**Figure 4:**
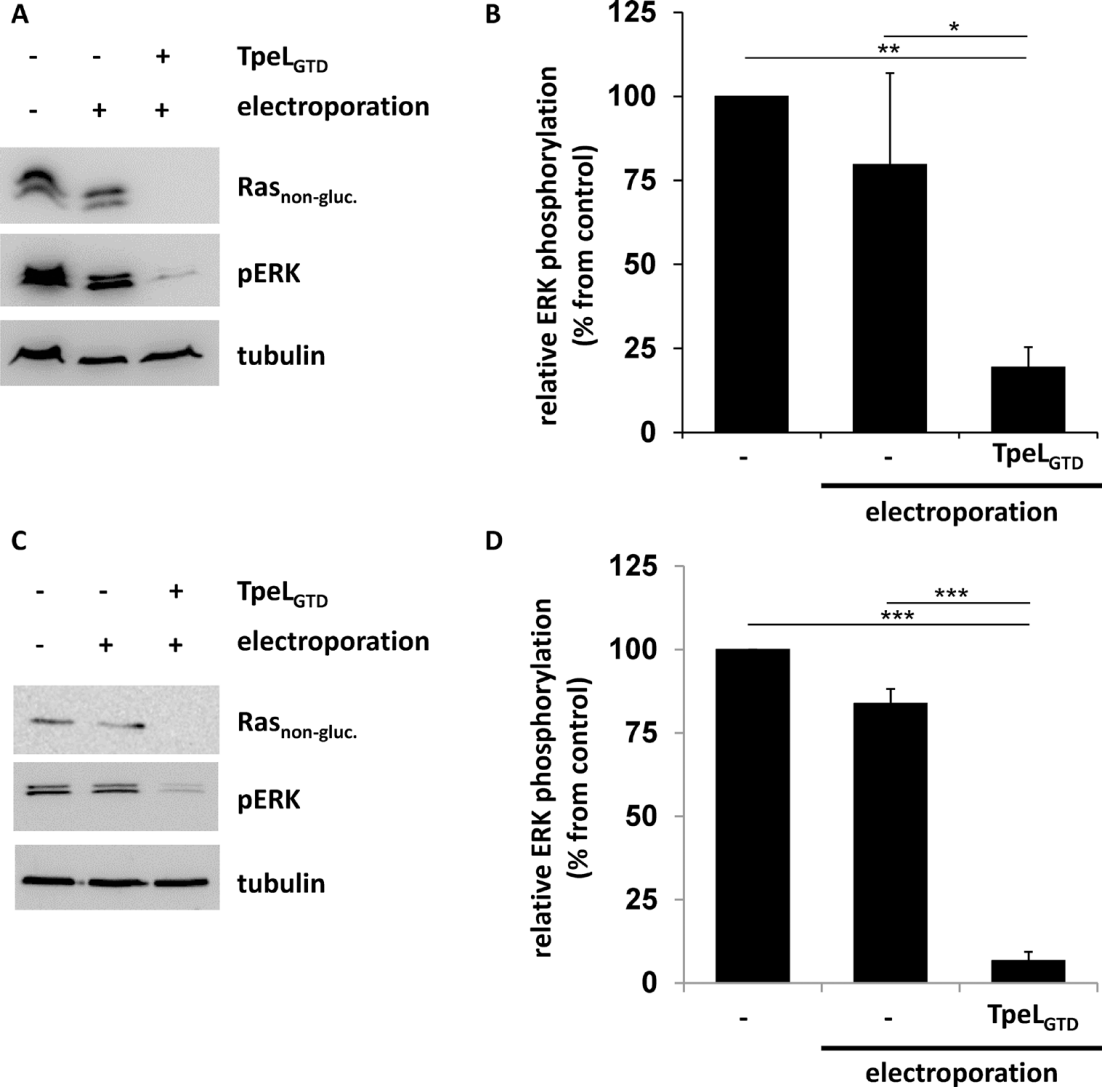
TpeL_GTD_ translocated into the cytosol of host cells by electroporation is sufficient to inactivate Ras (**A**) HCT-116 cells were electroporated in the presence of TpeL_GTD_ and plated for 2 h before lysis. Ras_non-gluc._, pERK, and tubulin were probed and a representative blot of three independent experiments is shown. (**B**) Statistical analysis of phosphorylated ERK following electroporation treatment as presented in A. (**C**) Capan-2 cells were electroporated in the presence of TpeL_GTD_ and plated for 2 h before lysis. Ras_non-gluc._, pERK, and tubulin were probed and a representative blot of three independent experiments is shown. (**D**) Statistical analysis of phosphorylated ERK following electroporation treatment as presented in C.

Studies with TpeL showed that some cell types were not sensitive towards toxin treatment, most likely, because the cellular toxin receptor and/or co-receptors are missing on their surface. One example are human colorectal cancer HCT-116 cells, which harbor a heterozygous *KRAS* G13D mutation [24]. Here, we aimed to treat the cells by electroporation in the presence of the glucosyltransferase domain of TpeL (TpeL_GTD_). After electroporation, cells were allowed to recover for 2 h. Thereafter, cells were lysed and analyzed for non-glycosylated Ras and phosphorylated ERK (pERK) by immunoblotting. Electroporation *per se* did not significantly decrease ERK phosphorylation (Figure 4A and 4B). After electroporation in the presence of the glucosyltransferase domain of TpeL, phosphorylated ERK (pERK) was significantly reduced (Figure 4) and the level of non-glycosylated Ras dropped below the detection limit. Similar results were obtained with the Capan-2 cell line derived from pancreas head ductal carcinoma, which carries a *KRAS* G12V mutation [25]. These cells are insensitive towards TpeL holotoxin, which is applied to the culture medium. However, electroporation in the presence of TpeL_GTD_ strongly reduced levels of unmodified Ras and pERK in Capan-2 cells (Figure 4C and 4D).

**Figure 5:**
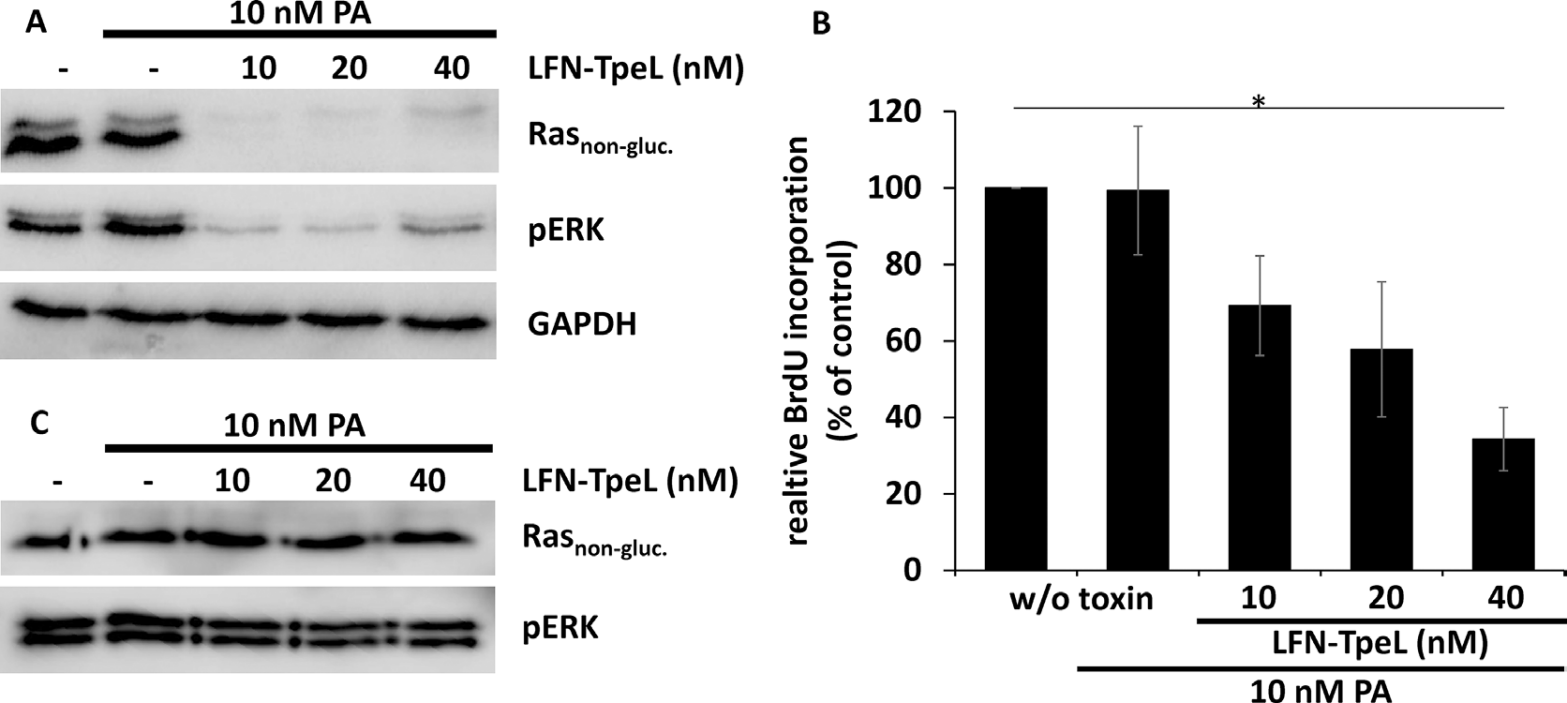
The glucosyltransferase domain of TpeL is transported effectively via PA into Panc-1 but not into Capan-2 cells (**A**) LFN-TpeL inhibits the Ras pathway in Panc-1 cells. Cells were intoxicated for 6 h with the indicated concentrations of LFN-TpeL and a fixed concentration of PA (10 nM). (**B**) The Western blot is a representative of three independent experiments. LFN-TpeL inhibits proliferation of Panc-1 cells. Cells were intoxicated as in A. Cell proliferation was measured by the incorporation of BrdU in intoxicated cells compared to an untreated control. The figure shows the result of three independent experiments. (**C**) LFN-TpeL is not effective in Capan-2 cells. Cells were intoxicated for 4 h at the indicated concentrations of LFN-TpeL and a fixed concentration of PA (10 nM). The Ras downstream signaling was not altered after intoxication. Data shown are a representative of three independent experiments.

Next, we made use of a transport system based on the protective antigen (PA), the binding and transport unit of anthrax toxin [24, 25]. PA forms heptamers following proteolytic activation and inserts into membranes at the low pH of endosomes. Thereby, a pore is formed that allows the transport of anthrax toxin enzymes (lethal factor and edema factor) or of artificial fusion proteins into mammalian cells. We generated a fusion protein composed of the N-terminal 263 amino acids of anthrax lethal factor (LFN) and the GTD of TpeL (LFN-TpeL). This part of LFN has no catalytic activity. It interacts with the PA heptamer and allows the transport of the GTD of TpeL through the PA pore. We tested the recombinant fusion toxin in pancreas adenocarcinoma cells Panc-1 and Capan-2 by co-incubation with PA. Cells were lysed and the lysates tested for Ras, modified Ras, activated ERK (pERK), and GAPDH as loading control. As shown in Figure 5A, a 10 nM concentration of the artificial toxin was sufficient to effectively modify Ras and to block Ras-signaling in Panc-1 cells. Additionally, we studied proliferation of toxin-treated cells. As shown in Figure 5B, proliferation was drastically reduced in the presence of 10 nM concentrations of each PA and LFN-TpeL. These data indicate that LFN-TpeL effectively modifies Ras and inhibits Ras signaling, resulting in inhibition of the proliferation of pancreatic cancer cells. However, we did not observe any effects of LFN-TpeL and PA on Capan-2 cells, even not at high concentrations (Figure 5C), indicating that the anthrax PA transport system is not efficient on Capan-2 cells.

**Figure 6:**
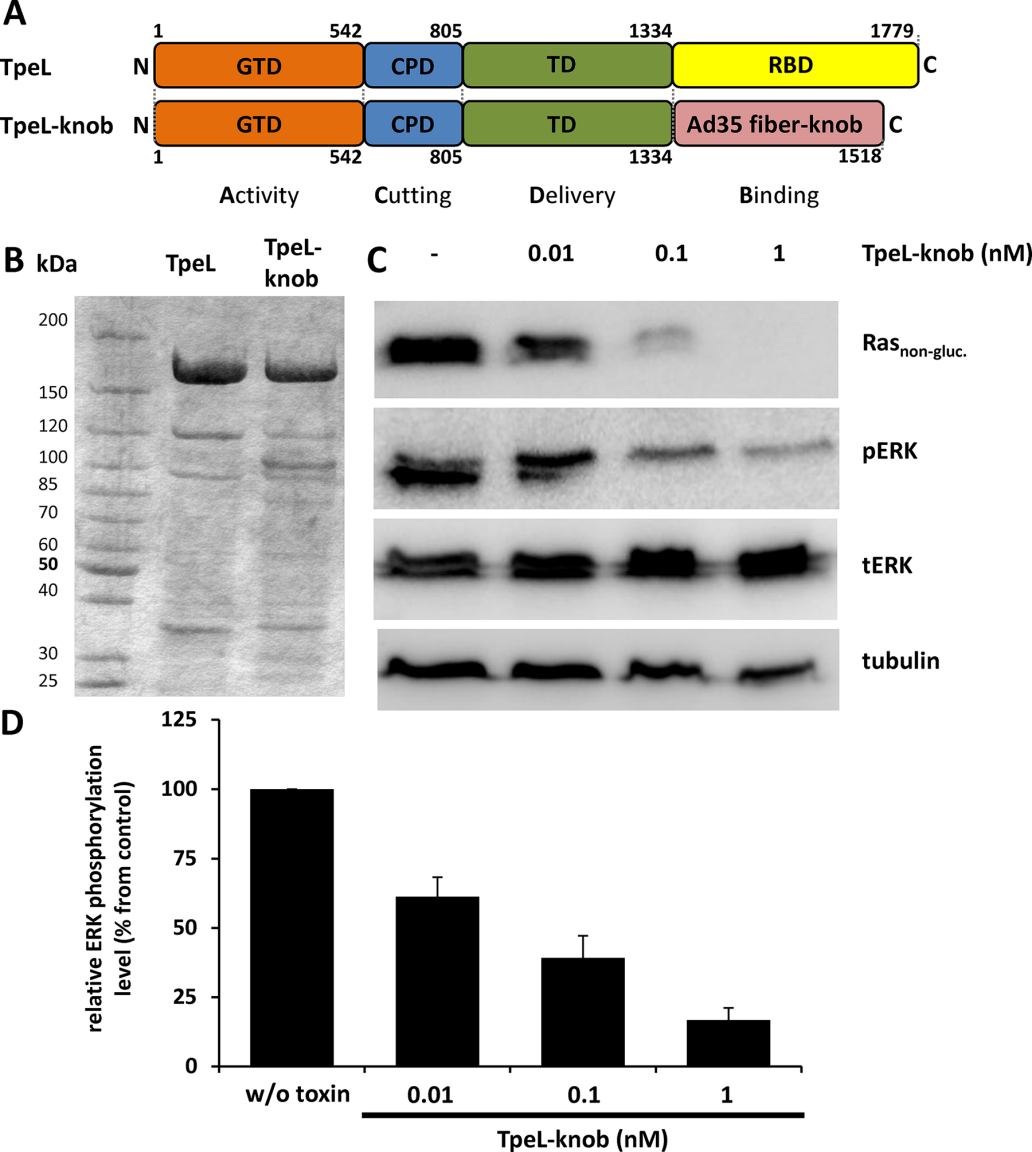
TpeL_GTD_ is targeted by the fiber knob to CD46-positive cell line Capan-2 (**A**) Schematic representation of the domain architecture of TpeL in comparison to the newly created fusion protein TpeL-knob. GTD, glycosyltransferase domain; CPD, cysteine protease domain; TD, translocation domain; RBD, receptor binding domain. (**B**) Coomassie-stained SDS-PAGE gel comparing the purity of TpeL and TpeL-knob proteins used in this study. (**C**) Western blot of Capan-2 cell lysates probed for Ras_non-gluc._, pERK, tERK, and tubulin. Prior to lysis cells were treated with increasing concentrations of TpeL-knob for 16 h. A representative blot of three independent experiments is shown. (**D**) Statistical analysis of the amount of phosphorylated ERK following toxin treatment as presented in C.

To further improve the potency and specificity of TpeL towards Capan-2 cells, we constructed a chimeric TpeL toxin with a changed receptor specificity. Capan-2 cells transcribe high levels of mRNA of the membrane protein CD46 [26]. CD46 is a complement inhibitor membrane cofactor, which also functions as a receptor for various microbes, including species B adenoviruses (Ad) serotype 35 [27, 28]. The knob region of fiber proteins of Ad35 specifically interacts with CD46 [29]. We constructed a chimeric TpeL fusion protein in which the native LRP1 binding region (amino acids 1334–1779) was deleted and changed for the Ad35 fiber knob domain. Figure 6A depicts the structure of the designed fusion protein (named TpeL-knob). TpeL-knob was expressed in *B. megaterium* as a 6xHis-tagged protein, purified and tested for cytotoxicity against tumor cells in cell culture. To test whether TpeL-knob was able to enter Capan-2 cells and to modify Ras, cells were challenged with increasing concentrations of the chimeric fusion toxin for 16 h. Figure 6C and 6D show that the fusion toxin reduced the levels of non-glycosylated Ras and pERK in a concentration-dependent manner. As provided in Supplementary Figure 2, unmodified TpeL was not able to induce changes in Ras signaling of Capan-2 cells. On the other hand, TpeL-knob fusion toxin was highly specific for tumor cells, because primary Normal Human Dermal Fibroblasts (NHDF) of the foreskin, which reportedly express no or very low amounts of CD46 [30] were not affected by the fusion toxin, while wild-type TpeL readily modified Ras of these cells (Supplementary Figure 3). Thus, both targeting approaches (i.e., PA-dependent LFN-TpeL transport and CD46-dependent up-take of TpeL-knob) confirmed as proof-of-concept experiments the anti-Ras activity of chimeric fusion toxin with TpeL_GTD_.

## DISCUSSION

TpeL causes GlcNAcylation of Ras proteins at threonine35 thereby inactivating these important switch proteins, which are involved in several fundamental cellular processes controlling proliferation, differentiation and survival [3]. Activated mutant Ras proteins are one of the most important oncogenic drivers, which are found in numerous cancer cells. Some cancer types exhibit a very high degree of Ras mutations, including pancreatic cancers, harboring in 95% of cases oncogenic Ras mutations [31–33]. Despite this obvious importance in carcinogenesis, development of therapeutic Ras inhibitors was not successful so far. Therefore, inactivation of Ras by TpeL-induced GlcNAcylation is of major interest. Here, we show that all major Ras isoforms are substrates for TpeL (see also [10]). Moreover, the inactive as well as the active forms of Ras were modified by TpeL. This finding is not only important for a later use to target hyperactive Ras proteins, it was additionally highly unexpected as a closely related family member of the large glycosylating toxins, the lethal toxin of *Clostridium sordellii* strongly favors Ras proteins in the inactive GDP-bound state as a substrate [34]. Moreover, different Ras mutants, frequently found in tumors, were also modified by TpeL. In cell culture, TpeL blocked at subnanomolar concentrations Ras and Ras-dependent MAP kinase activation. Of special interest is the finding that the Vemurafenib-induced paradoxical activation of the MAP kinase pathway in cells [19, 20], which do not harbor a *BRAF* V600E mutation, was blocked by TpeL. Considering that TpeL blocks Ras but no other components of the MAP kinase pathways, the data support the view that Ras activation is essential for Vemurafenib-induced paradoxical activation of MAP kinases, which might be caused by a transactivation and formation of B-Raf homo- or heterodimers [19, 20]. Therefore, the TpeL data complement previous studies showing that point mutations, abrogating the function of the Ras-binding domains of C-Raf (R89L) and B-Raf (R188L), prevent Raf hetero-dimerization and paradoxical MEK/ERK pathway activation [18, 22].

Our studies with zebrafish embryos, overexpressing human *HRAS* G12V in melanocytes and developing melanoma at one month of age [23], show that this model can be used to assess the effects of TpeL *in vivo*. Zebrafish are highly susceptible to TpeL toxin, indicating that zebrafish cells contain LRP1 receptor homologs. Here, fish embryos were employed, which overexpressed mutant Ras protein under the control of the melanocyte-specific *kita* promotor [23]. It is remarkable that the toxin exhibited very drastic effects on melanocyte proliferation and mutant Ras signaling, while the development of the embryos was not affected. Therefore, this animal model appears to be of high value for the study of the anti-Ras activity and general toxicity of TpeL.

TpeL binds to target cells via LRP1, which is present on many cell types [15, 35]. We observed that some cell types (e.g., HCT-116 and Capan-2 cells) were insensitive towards TpeL. However, TpeL was active in these cells after electroporation of the glycosyltransferase domain alone. Therefore, we decided to make use of the protective antigen PA of anthrax toxin to deliver the GTD of TpeL into target cells. To this end, a chimeric toxin of the GTD of TpeL with the N-terminal adaptor domain of LFN was constructed. This LFN part interacts with PA but possesses no enzyme activity. By means of PA, LFN-TpeL was taken up by Panc-1 cells and efficiently caused glycosylation of Ras with inhibition of ERK phosphorylation and inhibition of proliferation. However, it showed no influence on Capan-2 cells. Therefore, a different approach was performed to transport TpeL into Capan-2 cells: We replaced the receptor binding domain of TpeL, without changing the Ras-inactivating glucosyltransferase activity, the endogenous auto-protease activity and the translocation properties of the toxin. Paradigmatic for this approach are immunotoxins on the basis of diphtheria toxin and Pseudomonas exoenzyme A [36–40]. We engineered the receptor binding site of TpeL and changed the region, which is involved in interaction with LRP1 to the knob region of adenovirus serotype 35 fiber protein [29]. The knob region is essential for the interaction of the virus with host cells, where it is a ligand of the membrane protein CD46, which is frequently overexpressed in tumor cells [41–43]. Notably, the knob domain has been already used in the targeting of viral gene therapy with antitumor activity [30, 44, 45]. Here, the TpeL-knob fusion protein was tested on the human pancreatic tumor cell line Capan-2. The cells were treated with the fusion protein and MAP kinase signaling activity was determined. Following 16 h of incubation with 1 nM TpeL-knob, we were not able to detect any non-glycosylated Ras. In addition, the phosphorylation of ERK was strongly reduced. These findings indicate that knob-modified TpeL can enter cells and is catalytically active in the cytoplasm. Moreover, the TpeL-knob fusion protein was specific for tumor cells. Treatment of NHDF, which apparently express no or only very low amounts of CD46 on the cell surface [30], were insensitive towards the TpeL-knob fusion-toxin, but were intoxicated by wild-type TpeL. To further improve the uptake and effectiveness of knob-modified TpeL, mutations might be introduced into the knob sequence. Wang and coworkers described two mutant knob variants with highly increased binding to CD46 for virus targeting [46]. It would be of interest to test, whether these modifications in the TpeL-knob fusion toxin increase the potency of the TpeL-knob fusion toxin to inactivate Ras in tumor cells.

Taken together, our studies show that *C. perfringens* toxin TpeL GlcNAcylates all main types of oncogenic Ras proteins *in vitro* and *in vivo*. Toxin-catalyzed modification of Ras is accompanied by inhibition of the MAP kinase pathway. These toxin effects are also observed in a zebrafish model, expressing oncogenic Ras. Application of potent toxins as pharmacological tools or as drugs is often hampered by the fact that the toxins’ effects are not specific. In most cases, specificity of toxins is defined by receptor binding. We show that TpeL can be introduced into target cells by the binding and delivery component PA of anthrax toxin. Moreover, in a proof of principle study, aiming the construction of an anti-Ras immunotoxin, we demonstrate that the target-cell specificity of TpeL can be successfully changed. Of special interest is the successful introduction of the knob region of adenovirus serotype 35 fiber as a receptor binding site, which has not been successfully used for a toxin-chimeric protein before. Bacterial toxins like TpeL might offer a new direction for the search of Ras-inactivating agents. In line with this notion are recent findings, showing that the DUF5 domain of the *Vibrio vulnificus* MARTX toxin inhibits Ras signaling by specific cleavage of the small GTPase in the switch-I-domain [47].

## MATERIALS AND METHODS

### Cell cultivation and intoxication

Capan-2, HCT116, and Panc-1 cell lines were obtained from ATCC by LGC Standards GmbH (Wesel, Germany). SBCL2 cells were a kind gift of Dr. Meenhard Herlyn (Wistar Institute, Philadelphia, USA) and Primary Normal Human Dermal Fibroblasts (NHDF) were provided by Dr. Melanie Börries (ZBMZ, Freiburg, Germany). Cells were cultivated at 37°C with 5% CO_2_ under humidified conditions. Only cells that were tested negative for mycoplasma contamination were taken in experiments. Capan-2 cells were grown in RPMI1640 containing 15% FCS, sodium pyruvate, and non-essential amino acids. HCT116, NHDF, and Panc-1 cell medium was DMEM with 10% FCS, sodium pyruvate, and non-essential amino acids. SBCL2 medium was a mixture of MCDB153 with 20% Leibovitz’s L-15, 2% FCS, 5 µg/ml insulin, and 1.7 mM CaCl_2_. All cell media were supplemented with penicillin/streptomycin. All intoxication experiments were performed in fresh medium at standard culture conditions with the indicated amounts of toxin/protein for the indicated time. Treatment was stopped by removing the medium and washing cells with ice-cold PBS. Vemurafenib was from Selleck Chemicals (Houston, Texas, USA) and used for pre-incubation followed by intoxication as indicated.

### Protein expression and fusion protein cloning

Full-length TpeL (strain JGS1495) and TpeL-knob fusion protein were used in this study as recombinant, C-terminally His-tagged proteins. Cloning of the toxin gene into the bacterial expression vector pHIS1522, expression in the expression host *Bacillus megaterium,* and nickel affinity purification of the recombinant toxins were described before [10]. TpeL-knob was cloned by amplification of the fiber knob domain of adenovirus 35 (pQE30-Ad35knob, a kind gift from André Lieber, Division of Medical Genetics, University of Washington, Seattle, Washington, USA) with the following primers: sense primer 5′-ATGGTACCATGGATCCGGTGACATTTG and antisense primer 5′-ATGGTACCTGTT GTCTTCTGTAATGTAAG. By *Kpn*I restriction digest and ligation, this fragment was fused to the 3’-end of a pHIS1522-TpeL construct ending with amino acid 1334. For LFN-TpeL, the N-terminal 263 amino residues of LF were amplified with following primers: sense primer 5′-CATATGGCGGGCGGTCATGGTGA and antisense primer 5′-GGATCCCCGTTGATC TTTAAGTTCTTCCAAGG. The PCR product with flanking *Nde*I and *Bam*HI restriction sites was then cloned into the TpeL^1-542^-pET-28a (+) vector and verified by sequencing. Recombinant wild-type and mutant Ras proteins were expressed in *E. coli* with an N-terminal GST-tag as described by [48]. PA and LFN-TpeL were expressed with a His-tag as described before [49].

### Electroporation of mammalian cells

HCT116 or Capan-2 cells were counted and adjusted to 5 × 10^6^ cells per ml. The cell suspension was supplemented with 20 nM TpeL_GTD_ or vehicle control. 800 µl per condition were electroporated in a 0.4 mm cuvette with a GenePulser II (Biorad, Munich, Germany) using a capacitance of 250 μF and a voltage of 330 V. After addition of 1200 µl cell culture medium cells were allowed to recover for 2 h under standard cell culture conditions. Attached cells were resuspended by scratching to collect all cells. Eventually, cells were spun down and washed once with ice cold PBS before lysis and immunoblotting.

### *In vitro* glycosylation assay

Recombinant Ras proteins (10 µM) were incubated with 10 nM TpeL together with 10 µM UDP-[^14^C]-N-acetyl-glucosamine (Biotrend, Cologne, Germany) in a buffer containing 50 mM HEPES at pH 7.5, 0.1 M KCl, 2 mM MgCl_2_, 1 mM MnCl_2_, and 0.1 mg/ml BSA. Glycosylation was performed for 10 and 20 min at 37 °C and stopped by the addition of SDS-containing sample buffer and boiling. Samples were subjected to SDS-PAGE, dried gels were autoradiographed, and transferred sugar moieties quantified (PhosphoImager SI, GE Healthcare). If indicated, K-Ras was preloaded with GDP, GTPγS or a mixture of GDP and AlF_3_ prior to glycosylation essentially as described by [50].

### Cell lysis and immunoblotting

Whole-cell lysates were prepared by incubation on ice in lysis buffer containing 50 mM Tris at pH 7.5, 1% Triton X-100, 0.5% sodium deoxycholate, 0.1% SDS, 137 mM NaCl, 1 mM Na_3_VO_4_, and complete protease inhibitor mix (Roche, Mannheim, Germany). Cell remnants were removed by centrifugation (21,000 × g, 10 min, 4°C), and the protein concentration of the protein lysate was estimated by a bicinchoninic acid assay kit (Uptima, Montluçon Cedex, France). Proteins in the lysate were separated by SDS-PAGE and transferred onto a PVDF membrane by western blotting for antigen detection by chemiluminescence using the LAS-3000 reader (FujiFilm, Dusseldorf, Germany). Non-glycosylated Ras (Ras_non-gluc_.), phospho-ERK (pERK), total ERK (tERK), phospho-MEK (pMEK), and tubulin were detected with the primary antibodies rabbit anti-Ras (27H5) (3339; Cell Signaling, Leiden, Netherlands), rabbit anti-phospho-p44/42 MAPK (ERK1/2) (Thr202/Tyr204) XP (4370; Cell Signaling), anti-p44/42 MAPK (ERK1/2) (4695; Cell Signaling), rabbit anti-phospho-MEK1/2 (Ser217/221) (9154, Cell Signaling), and mouse anti–α-tubulin (T9026; Sigma-Aldrich), respectively. Horseradish peroxidase-conjugated donkey anti-mouse IgG (H&L) (610-703-124; Rockland Scientific, Victoria, Canada) or goat anti-rabbit IgG (H&L) (7074; Cell Signaling) antibodies were used as secondary antibodies. Antibody signals were visualized by the enhanced chemiluminescence reaction and quantified with MultiGauge software (FujiFilm, Dusseldorf, Germany). Cropping, as well as contrast and brightness adjustment were performed for whole blots only.

### Proliferation assay

Panc-1 cells were seeded in a 96 well plate overnight then intoxicated with the indicated concentrations of PA and LFN-TpeL for 48 h. The proliferation was measured using the chemiluminescent cell proliferation ELISA BrdU assay (Roche). BrdU was added to each well according to the manufacturer´s protocol for 150 min. Cells were fixed for 30 min with the provided cell fixation solution. Anti-BrdU-peroxidase solution was added for 90 min and cells were washed three times with a washing solution and developed using the substrates provided by the kit for 3 min. The signal was measured on a Tecan Infinite M 200.

### Zebrafish handling

Zebrafish experiments were performed under EU regulations for animal experimentation with the permission of the federal state of Baden-Württemberg, Germany. Embryos used were reared at 28.5°C on a 14/10 h light/dark cycle in E3 medium. The generation of kita-GFP-RAS zebrafish was described before [23]. Eggs were dechorionated 6 h post-fertilization (hpf) and treatment with 30 nM TpeL/0.5% DMSO was initiated at 12 hpf. Toxin solution was replaced at 36 hpf and larvae were subjected to microscopy at 60 hpf. Alternatively, 20 larvae per condition were pooled, deyolked, and subjected to cell lysis and immunoblotting.

### Statistics

Significance differences between sample groups were calculated in Sigmaplot by pairwise comparison using the one-way ANOVA test (Holm-Sidak method). Resulting *p* values were indicated by asterisks as follows: ^*^p < 0.05, ^**^p < 0.01, ^***^p < 0.005. Error bars represent the standard error of the means (SEM).

## SUPPLEMENTARY MATERIALS FIGURES


