# Supplementary material for: Targeting oncogenic Ras by the Clostridium perfringens toxin TpeL
Source: Oncotarget. 2018 Mar 27;9(23):16489–500. doi: 10.18632/oncotarget.24740 (PMC5893256; doi:10.18632/oncotarget.24740)
Supplement: Supplementary file 1 [file oncotarget-09-16489-s001.pdf]

Targeting oncogenic Ras by the *Clostridium perfringens* toxin TpeL

SUPPLEMENTARY MATERIALS

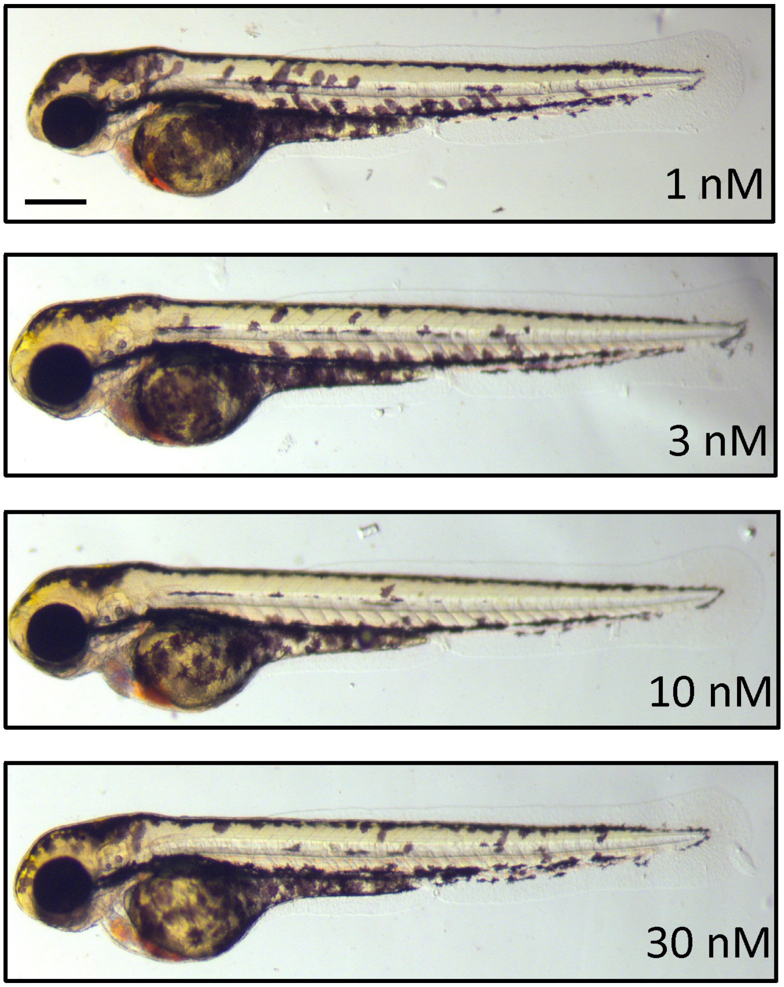

Supplementary Figure 1: Zebrafish embryos treated with increasing concentrations of TpeL show no signs of toxicity. Micrograph of zebrafish larvae with Ras-dependent hyperpigmentation at 60 hpf treated with increasing concentrations of TpeL from 12 hpf on. Beside the reduced number of melanocyte no changes in morphology were observed, suggesting the lack of major toxic effects. Representative fish of 20 larvae per condition are shown as an extension to Figure 3A and the scale bar represents 0.1 mm.

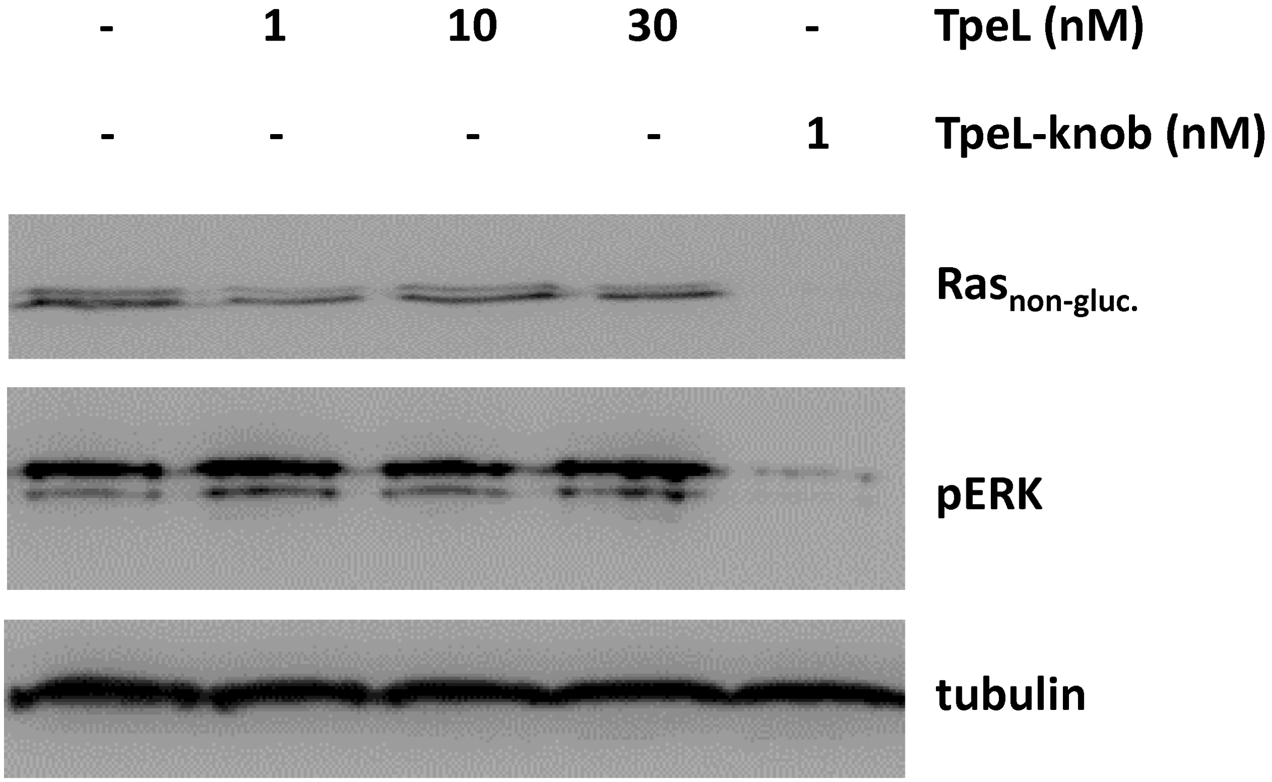

Supplementary Figure 2: TpeL does not intoxicate the cell line Capan-2 in the wild type form. Western blot of Capan-2 cell lysates probed for $Ras_{non\text{-}gluc.}$, pERK, and tubulin. Prior to lysis cells were treated with increasing concentrations of TpeL or TpeL-knob (as a positive control) for 16 h.

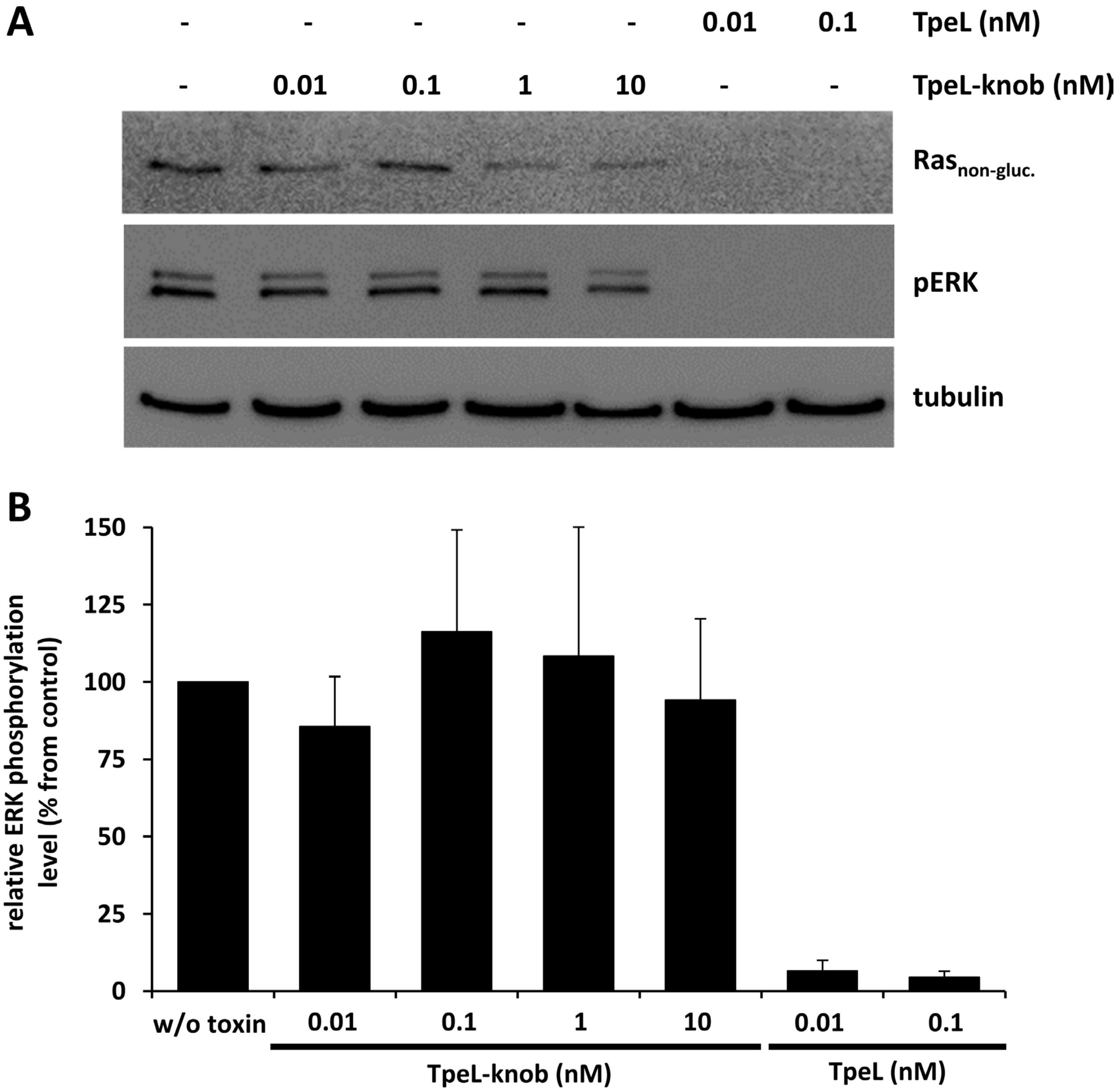

Supplementary Figure 3: TpeL, but not TpeL-knob, intoxicates primary human dermal fibroblasts (NHDF) of the foreskin. (**A**) Western blot of NHDF lysates probed for $Ras_{non\text{-}gluc.}$, pERK, and tubulin. Prior to lysis cells were treated with increasing concentrations of TpeL-knob or wild-type TpeL for 16 h. A representative blot of three independent experiments is shown. (**B**) Statistical analysis of the amount of phosphorylated ERK following toxin treatment as presented in A.
